# Ecosystem Services and Opportunity Costs Shift Spatial Priorities for Conserving Forest Biodiversity

**DOI:** 10.1371/journal.pone.0112557

**Published:** 2014-11-13

**Authors:** Matthias Schröter, Graciela M. Rusch, David N. Barton, Stefan Blumentrath, Björn Nordén

**Affiliations:** 1 Environmental Systems Analysis Group, Wageningen University, Wageningen, The Netherlands; 2 Norwegian Institute for Nature Research (NINA), Trondheim/Oslo, Norway; Chinese Academy of Forestry, China

## Abstract

Inclusion of spatially explicit information on ecosystem services in conservation planning is a fairly new practice. This study analyses how the incorporation of ecosystem services as conservation features can affect conservation of forest biodiversity and how different opportunity cost constraints can change spatial priorities for conservation. We created spatially explicit cost-effective conservation scenarios for 59 forest biodiversity features and five ecosystem services in the county of Telemark (Norway) with the help of the heuristic optimisation planning software, Marxan with Zones. We combined a mix of conservation instruments where forestry is either completely (non-use zone) or partially restricted (partial use zone). Opportunity costs were measured in terms of foregone timber harvest, an important provisioning service in Telemark. Including a number of ecosystem services shifted priority conservation sites compared to a case where only biodiversity was considered, and increased the area of both the partial (+36.2%) and the non-use zone (+3.2%). Furthermore, opportunity costs increased (+6.6%), which suggests that ecosystem services may not be a side-benefit of biodiversity conservation in this area. Opportunity cost levels were systematically changed to analyse their effect on spatial conservation priorities. Conservation of biodiversity and ecosystem services trades off against timber harvest. Currently designated nature reserves and landscape protection areas achieve a very low proportion (9.1%) of the conservation targets we set in our scenario, which illustrates the high importance given to timber production at present. A trade-off curve indicated that large marginal increases in conservation target achievement are possible when the budget for conservation is increased. Forty percent of the maximum hypothetical opportunity costs would yield an average conservation target achievement of 79%.

## Introduction

The ecosystem services (ES) concept comprises multiple contributions of ecosystems to human well-being [1], and has increasingly been used to raise awareness about the benefits that people derive from ecosystems [2], [3]. Considering ES when making decisions about the use of ecosystems could provide additional, anthropocentric arguments to support either management aimed at sustainable use of ecosystems or biodiversity conservation [4]. However, there is a still unresolved debate about to what extent components of biodiversity correspond with ES provision [4]–[7] and about the extent to which considering ES in decision making matches with biodiversity conservation objectives. Furthermore, accounting for ES within conservation planning is a fairly new practice [8]–[11]. In a conservation decision-making context, ES can be seen as benefits of conservation (many cultural and regulating services), or in the case of extractive provisioning services as an opportunity cost of conservation since their use may become restricted [8]. Trade-offs between extractive provisioning services, such as clear-cutting timber harvest, and other ES [12] and biodiversity protection [11], [13]–[15] require choices to be made on whether and where to protect an area. However, certain management systems restrict timber production and might thus allow for a synergy between an extractive provisioning service and other ecosystem services [16], [17] as well as some aspects of biodiversity conservation [17]–[21]. This leads to the crucial question within cost-effective conservation planning on how multiple-use areas, in which extractive exploitation is restricted, can potentially contribute to biodiversity conservation [22]–[24]. Cost-effective conservation means minimizing opportunity costs in terms of foregone commodity production [25]. As some conservation targets are compatible with a certain level of use [26], and since the opportunity costs of setting aside areas can be potentially high, a mixture of fully protected areas and areas allowing for partial use is likely to render more cost-effective and less conflictive conservation solutions, and may open opportunities for overall higher levels of biodiversity protection.

Spatial considerations play an integral role in the assessment of cost-effectiveness of conservation as the spatial configurations of important habitats [27] and of opportunity costs of conservation do not necessarily coincide [28]. A ‘policyscape’ may be defined as the spatial configuration of a mix of policy instruments [29], which aims at conserving biodiversity and ES at an aggregated spatial level. This framing suggests that there is an optimal and complementary spatial allocation of different types of instruments across a space containing all possible combinations of conservation values and opportunity costs within a study area. The spatial configuration of the policyscape has important practical implications for decision-making. For instance, it opens opportunities to evaluate disproportionate economic burdens between administrative units.

In this study, we suggest ways of creating cost-effective policyscapes. We address a mix of instruments that combines non-use (strict protection) and partial use (forestry restricted) for the conservation of forest biodiversity and ES in the county of Telemark (Norway). Indicators of the state of forests in Norway show a decline of certain species populations, especially of species associated to old-growth forest and species whose habitats are threatened by current forestry practices [14], [30]. There is a need to modify and adapt current conservation policies to help secure portions of unprotected biodiversity as well as to halt the processes that lead to forest biodiversity loss [14], [30], [31]. One approach is to increase protected forest areas in Norway, particularly within the ecological zones that are most favourable for forestry production [31]. Currently, new nature reserves in Norway are mostly implemented through voluntary forest conservation schemes that are based on a negotiation between forest owners and conservation authorities in Norway [32]. The exploration of different policyscapes for conservation of biodiversity and ES can give guidance to support such conservation efforts.

We used the conservation planning software Marxan with Zones [33] for near-optimal selection of areas for cost-effective policyscapes on a county level. Some experience has been developed in applying (earlier versions of) Marxan to conservation optimisation with ES [8], [11], [34]–[37]. However, to our knowledge integrated targeting of both biodiversity and multiple ES within a policyscape with different levels of protection has not been systematically studied before.

We addressed the following specific questions. We first analysed how optimal conservation outcomes differ between two scenarios that either take into account biodiversity only (scenario 1) or a set of ES next to biodiversity (scenario 2). The outcome of both scenarios was measured in terms of spatial configuration, area protected, conservation target achievement, and opportunity costs.

Second, we assessed the trade-off between biodiversity and ES conservation goals and timber production. We analysed this relationship by constructing a production possibility frontier (PPF) [25], while considering timber production as a private good and the sum of biodiversity features and other ES as public goods. These public goods are either spared from timber production in the case of full protection or jointly produced with the private good in the case of partial protection. We compared current instrument targeting, i.e. the effectiveness of current reserves to achieve conservation targets set in our scenario, to a ‘benchmark’ defined as the cost-effective policyscape traced by the PPF [38], [39].

Third, we explored differences in conservation burden across administrative units. For this purpose, we calculated the expected opportunity costs of an optimal conservation outcome for each municipality in Telemark. Significant differences in conservation burden across municipalities would suggest potential efficiency gains with concomitant distributional consequences, which could justify considering the introduction of a conservation instrument such as ecological fiscal transfer schemes [40].

## Methods

### Study area

Telemark is a county in southern Norway with an area of 15,300 km^2^ and a population of about 170,000 people [41], concentrated mainly in the south-eastern part of the county. The climate varies across the region with temperate conditions in the south-east (Skien, average temperature January −4.0°C, July 16.0°C, 855 mm annual precipitation) and alpine conditions in the north-west (Vinje, January −9.0°C, July 11.0°C, 1035 mm) [42]. The southern part of Telemark is mainly covered by forest exploited by forestry activities as well as by large inland lakes, with few towns and a small agricultural area (247 km^2^, i.e. about 1.6% of the land area) [41]. The northern part is characterised by treeless alpine highland plateaus covered by bogs, fens and heathlands [43]. The forest landscape in Telemark is characterized by coniferous and boreal deciduous forest [43]. Important forest ecosystem services include moose hunting, free range sheep grazing and timber production [44]. In addition, forests of Telemark sequester and store considerable amounts of carbon, prevent snow slides and provide opportunities for recreational hiking and residential amenities [44]. In 2011, conservation areas comprised about 5.1% in national parks, 4.6% in landscape protection areas (both types cover mainly highland plateaus), as well as 1.7% in nature reserves [41]. As a result of forestry activities, the status of biodiversity in forests of Telemark shows relatively low values compared to other ecosystems and regions within Norway [14]. We conducted our analysis for the forest area within Telemark, however, as forest field mapping is lacking for a small south-eastern part of the county [45], this area was excluded from the analysis.

### Principle of Marxan with Zones

Marxan with Zones [33] builds on a heuristic optimisation algorithm that incorporates key principles of systematic conservation planning, including comprehensiveness, cost-effectiveness and compactness of the reserve system [46]. Marxan with Zones enables to consider zones with different levels of protection and thus spatial differences in costs, thereby allowing for planning and evaluation of policyscapes that include full and partial protection. Marxan with Zones requires a series of inputs, which are specified below.

### Data input Marxan with Zones

#### ES and biodiversity features and conservation targets

Depending on the scenario, a total of 59 (scenario 1, biodiversity) and 64 (scenario 2, biodiversity and ES) input features were used, respectively. Table 1 provides an overview of all features.

**Table 1:** Features, targets, fraction of targets to be achieved across the two zones (non-use and partial use), and contribution (effectiveness) of the partial zone in meeting respective targets.

Feature name	Feature target (%)	Fraction non-use (%)	Fraction partial (%) (contribution in %)
**Wilderness-like areas (ES)**	100	100	0 (0)
**Recreational hiking (ES)**	20	50	50 (100)
**Carbon storage (ES)**	10	50	50 (25)
**Carbon sequestration (ES)**	5.57	75	25 (25)
**Snow slide protection (ES)**	100	0	100 (100)
**Old-growth forest types (40)**	50	75	25 (50)
**Corridors (6)**	50	50	50 (50)
**Priority habitats for conservation (very important)**	100	100	0 (0)
**Priority habitats for conservation (important)**	100	100	0 (0)
**Priority habitats for conservation (locally important)**	50	100	0 (0)
**Hollow deciduous trees**	100	100	0 (0)
**Late successional forests with deciduous trees**	100	100	0 (0)
**Logs**	100	100	0 (0)
**Old trees**	100	100	0 (0)
**Rich ground vegetation**	100	100	0 (0)
**Snags**	100	100	0 (0)
**Trees with nutrient-rich bark**	100	100	0 (0)
**Trees with pendant lichens**	100	100	0 (0)
**Recently burned forest**	100	100	0 (0)
**Stream gorges**	100	100	0 (0)

We included five key ES of importance within a Norwegian context for which spatial models have been developed (Table 1) [44]. We specifically included biodiversity features which are characteristic of old-growth, largely undisturbed forest and which are not maintained under current commercial forestry practices. We included 40 types of old-growth forest, to a large extent remnants of previously high-graded forests, occurring across a range of vegetation zones, climate zones and productivity conditions to represent the ecological variability across the county (Text S1 for details). Six proposed forest corridors of national importance that connect existing reserves [47] were included as a spatial indicator of conditions enabling species dispersal between habitats [48]. Forest habitats of particular conservation importance on a national level in Norway [49], [50] were also included. Three classes of priority habitats for conservation (very important, important and locally important) were taken from the Norwegian Environmental Agency’s database (Naturbase) [51]. In addition, we included ten types of important forest habitats (Table 1) from a Norwegian Forest and Landscape Institute database (MiS) [52].

Marxan with Zones requires setting quantitative conservation feature targets that reflect the proportion of the abundance of each feature to be protected. Targets were based on expert judgments and, wherever possible, on interpretation of policy documents (Table 1, and Text S1 for details). In order to verify targets an expert workshop was organised (Text S1). Written consent to participate in this study was obtained from the participants of the expert workshop.

#### The policyscape – definition of zones, zone targets, zone contributions

Two types of area protection were included in our analysis, namely a non-use and a partial use zone. Non-use referred to nature reserves, where forestry is completely restricted, i.e. “use” refers to forestry activities. The partial use zone was an ‘umbrella’ zone covering three different current forms of protection where forestry is partially restricted, namely landscape protection areas, mountain forest (‘fjellskog’), and outdoor recreation areas (‘friluftsområder’) (Text S1). All current nature reserves in Telemark [51] were ‘locked-in’ as non-use zones and all current landscape protection areas were ‘locked-in’ as partial use zones, which means that spatial units overlapping with these areas were selected for the respective zone in each run of Marxan.

Marxan with Zones allows for distribution of the targets across zones. Zone targets were defined according to an own expert judgement about how well the non-use and partial use areas were compatible with the persistence of the respective feature. Zone targets (Table 1) were discussed, reviewed and as far as possible confirmed during the expert workshop (Text S1).

Marxan with Zones allows for differentiation of how effective zones are in order to achieve targets (zone contribution). We considered the effectiveness of partial use areas as “the relative contribution of actions to realizing conservation objectives” [53]. We assumed that non-use areas are fully effective to reach the targets of all features (100% contribution). There is growing, but yet inconclusive knowledge on how low impact logging could be compatible with biodiversity conservation [17]–[21], [54], [55]. This means that effectiveness of partial use areas is highly uncertain, and may affect features differently. Zone contributions were thus discussed and as far as possible confirmed during the expert workshop. In a sensitivity analysis we further explored the consequences of changing the zone contribution of the partial use zone (File S1).

#### Planning units

The forest area in Telemark was divided into 43.513 grid planning units of 25 ha size (500 m×500 m). This resolution was suitable in terms of time and computing capacity, and considered relevant for land-use planning. Property sizes in Norwegian forests vary widely from as little as 0.1 ha to several hundred hectares [32] and as such are not a good guide to setting the size of the planning unit.

#### Opportunity costs of conservation

Foregone timber harvest was selected as an indicator of opportunity costs of conservation since harvest activities are constrained by different forms of protection [25]. We used a net revenue (stumpage value) forest model to determine opportunity costs (Text S1). In non-use areas opportunity costs were set to 100%, while in partial-use areas, we estimated that restrictions would account for 25% of the stumpage value. This estimate was based on different logging restrictions [56] which ranged from 15% (landscape protection area), to 20% (outdoor recreation area) and 30% (mountain forest).

### Analyses

Marxan with Zones was run 20 times with the parameters described above (for further parameter adjustments see Table S1 and Table S2). The software was run for both scenarios to determine the best solution and the selection frequency of each planning unit over all runs, which ranged from 0 (never chosen) to the maximum of 20 (chosen in each run) and indicated importance of a particular planning unit to achieve the overall conservation targets [57]. Marxan with Zones input files, including spatial information on all conservation features, can be found in the supporting information for scenario 1 (File S2) and scenario 2 (File S3).

#### Comparison of scenarios

We used selection frequency of planning units to determine how the policyscapes of both scenarios differed spatially. Selection frequency of each planning unit to each of the two zones in scenario 1 (biodiversity only) was subtracted from selection frequency in scenario 2 (biodiversity and ES) to determine the difference. To compare the spatial configuration of the policyscapes, we calculated Pearson’s correlation coefficient between the selection frequency of each scenario for the partial and the non-use zone. We calculated Cohen’s Kappa on the selection frequency of each planning unit as a measure of agreement between the scenarios for each zone. To compare the two scenarios in absolute terms we calculated a number of statistics, including total costs, number of planning units without protection, planning units in the partial and non-use zone and average target achievement.

#### Trade-off between conservation target achievement and timber harvest

The PPF was identified by running a series of cost constraints for scenario 2. Cost constraints are a restricting condition that defines an upper limit of costs when selecting planning units. We started by running the scenario with no cost constraints and close to 100% average target achievement, and recorded the total unconstrained cost. We then introduced cost constraints at different levels (80%, 60%, 40%, 20%, 10%, 5%, 1%) of the total unconstrained cost in consecutive runs (see Table S4 for parameter details). The value of timber production (horizontal axis in the PPF) was determined as the total sum of stumpage value across all planning units in the study area minus the opportunity cost of the best solution of each run. The vertical axis in the PPF was determined as the average percentage of target achievement for all biodiversity and ES features. To assess the opportunity costs of conservation and the conservation target achievement of the current existing reserve network, we used an overlay analysis (r.stats in GRASS GIS).

#### Conservation burden across Telemark

To determine the conservation burden among the municipalities in Telemark, the expected opportunity cost for each municipality was calculated as the summed expected value of opportunity costs:
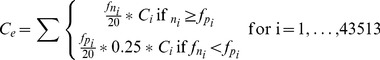
(1)where *C_e_* is the expected opportunity cost, *f_ni_* is the selection frequency of non-use areas for planning unit *i*, *f_pi_* is the selection frequency of partial use areas for planning unit *i* and *C_i_* is the opportunity cost of planning unit *i*. The denominator 20 stands for the number of runs in our case and the factor 0.25 specifies the harvest restriction in the partial use areas.

This analysis was run on scenario 2 with first, no cost constraint and, second, a medium cost constraint of 60% of the maximum costs needed to achieve close to 100% of the average targets. Opportunity costs per municipality were determined with zonal statistics in ArcMap for both expected opportunity cost layers and for current reserves. Municipalities were ranked according to relative opportunity costs, i.e. opportunity costs divided by municipal forest area. To analyse the spatial shift of the conservation burden across municipalities, Spearman’s rank correlation coefficient was calculated between the current situation and the unconstrained scenario, as well as between the 60% cost constraint and the unconstrained scenario.

## Results

### Incorporating ES in the policyscape for biodiversity conservation

**Figure 1:**
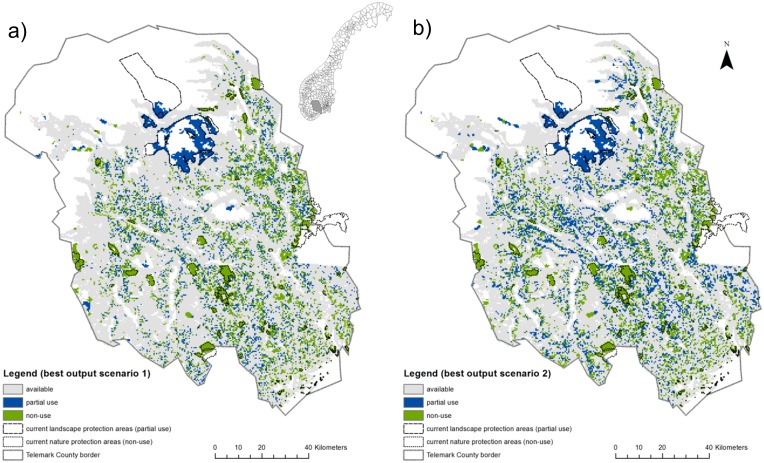
Best solution of the reserve network for scenario 1 (a) and scenario 2 (b). Scenario 1, considers biodiversity conservation criteria only; scenario 2, both biodiversity and ecosystem services criteria. Grey, areas available for forestry; blue, areas in the partial use zone and green, areas in the non-use zone. Current reserves are demarcated in dashed lines. Map inlay shows the location of Telemark within Norway (grey).

**Figure 2:**
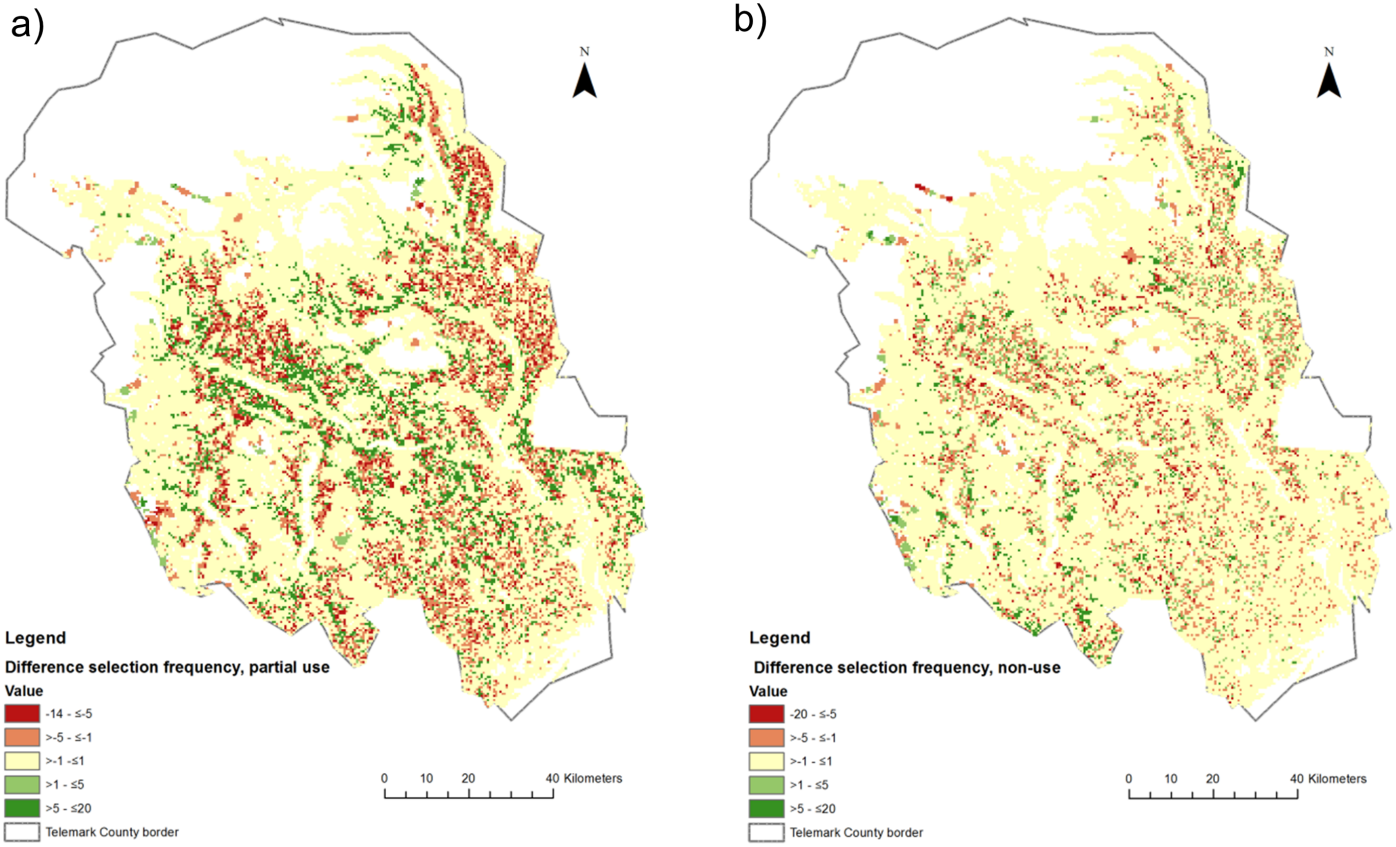
Differences in selection frequency of sites for partial (a) and non-use (b) areas. The maps show the difference of scenario 2 (biodiversity and ES features) versus scenario 1 (biodiversity only). A positive difference means higher selection frequency in scenario 2 than in scenario 1.

Incorporating ES into the policyscape changed the absolute sum of area in the two zones, the opportunity costs (Table 2) as well as the spatial configuration of the policyscape (Figures 1 and 2). When considering ES, the sum of partial use areas increased by 36.2% and the sum of non-use-areas by 3.2% compared to the scenario that only considered biodiversity. Opportunity costs were 6.6% higher in scenario 2 than in scenario 1. As an illustration of a policyscape, Figure 1 shows the best solution per scenario for scenario 1 (a) and scenario 2 (b). Selection frequencies of planning units for both scenarios can be found in Figure S1. The differences in selection frequencies are shown in Figure 2 for the partial (a) and non-use zone (b). A positive difference means higher selection frequency in the policyscape of scenario 2 than in scenario 1, while a negative difference indicates a lower selection frequency in the policyscape of scenario 2 than in scenario 1. Comparison of the spatial configuration of the policyscapes of both scenarios led to the following results. Pearson’s correlation coefficient between selection frequencies of sites in the non-use zone was r = 0.90, while for the partial use zone, it was r = 0.58. This indicates that relatively larger differences can be expected in the partial use zone than in the non-use zone when ES were considered, which partly rests upon the fact that ES can, in contrast to most of the biodiversity features in this study, partly be protected in this zone. Cohen’s Kappa statistics was K = 0.577 (sig≤0.0001) for the non-use zone and K = 0.398 (sig ≤0.0001) for the partial use zone. These results imply ‘moderate agreement’ in non-use and 'fair agreement’ in partial use zone, respectively [58], which supports the observation of a relatively larger agreement between non-use areas in the different spatial configurations of the policyscapes.

**Table 2:** Summary statistics describing the difference between scenario 1 (considering biodiversity conservation criteria only) and 2 (considering biodiversity and ecosystem services) in terms of opportunity costs, area in the different zones and average conservation target achievement.

Statistics	Scenario 1	Scenario 2	Difference 2 vs. 1 in %
**opportunity costs (billion NOK)**	1.912	2.038	+6.6
**without protection (no. of planning units of 25** **ha)**	32,183	30,279	−5.9
**partial use area (no. of planning units of 25** **ha)**	4,661	6,349	+36.2
**non-use (no. of planning units of 25** **ha)**	6,669	6,885	+3.2
**average conservation target achievement (%)**	99.86	99.23	−0.6

### Trade-offs between conservation and timber production: Production possibility frontier (PPF)

**Figure 3:**
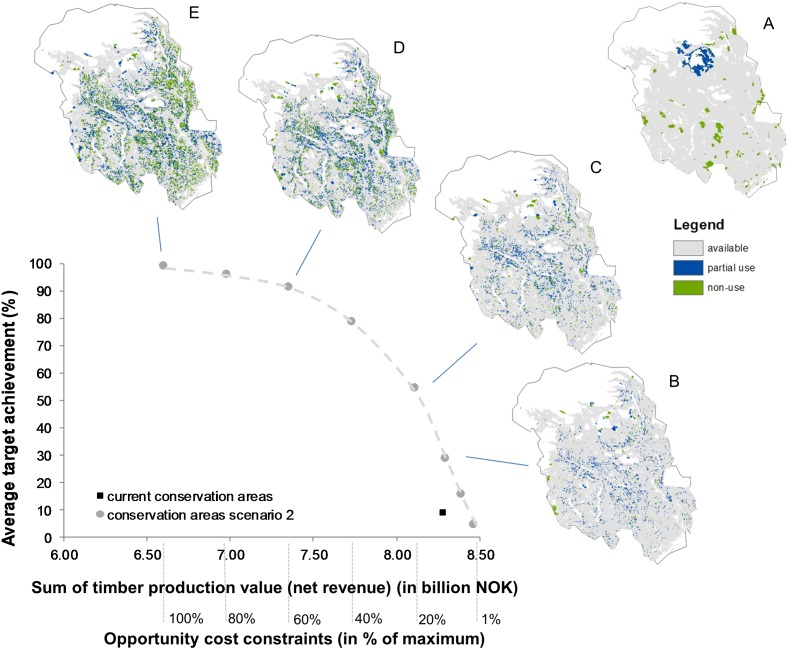
Forest conservation-timber production possibility frontier (PPF). Note that the x-axis (sum of timber production value) starts at 6.00 billion NOK. The maps indicate current reserve network (A) and selected (B–E) available, partial and non-use areas when current reserves are not locked-in. The spatially explicit solutions (policyscapes) are shown as maps on the trade-off between net revenues from timber production and average conservation target achievement, along a range of opportunity costs constraints.

The PPF shows a concave curve representing the trade-off between timber production and conservation of biodiversity and non-forestry related ES (Figure 3). Creating a reserve network to achieve the conservation targets comes at a cost of timber production. The marginal increase in conservation target achievement is initially high when the current constraint on conservation cost is relaxed (i.e. moving left in Figure 3). This marginal conservation gain decreases more rapidly after having passed a cost constraint of about 40% of the total cost required to achieve 100% of the overall conservation target. The current policyscape (black square) lies under the PPF curve, meaning that more cost-effective policyscape configurations than the current one are possible. This means that higher average target achievement could hypothetically be realised at current levels of timber production, or that the same target could be achieved at lower costs. At the same time, the location of the current policyscape shows a strong preference of decisions towards timber production. Consequently, the conservation targets we set in our scenario are barely met by the current reserve system (average achievement 9.1%).

**Figure 4:**
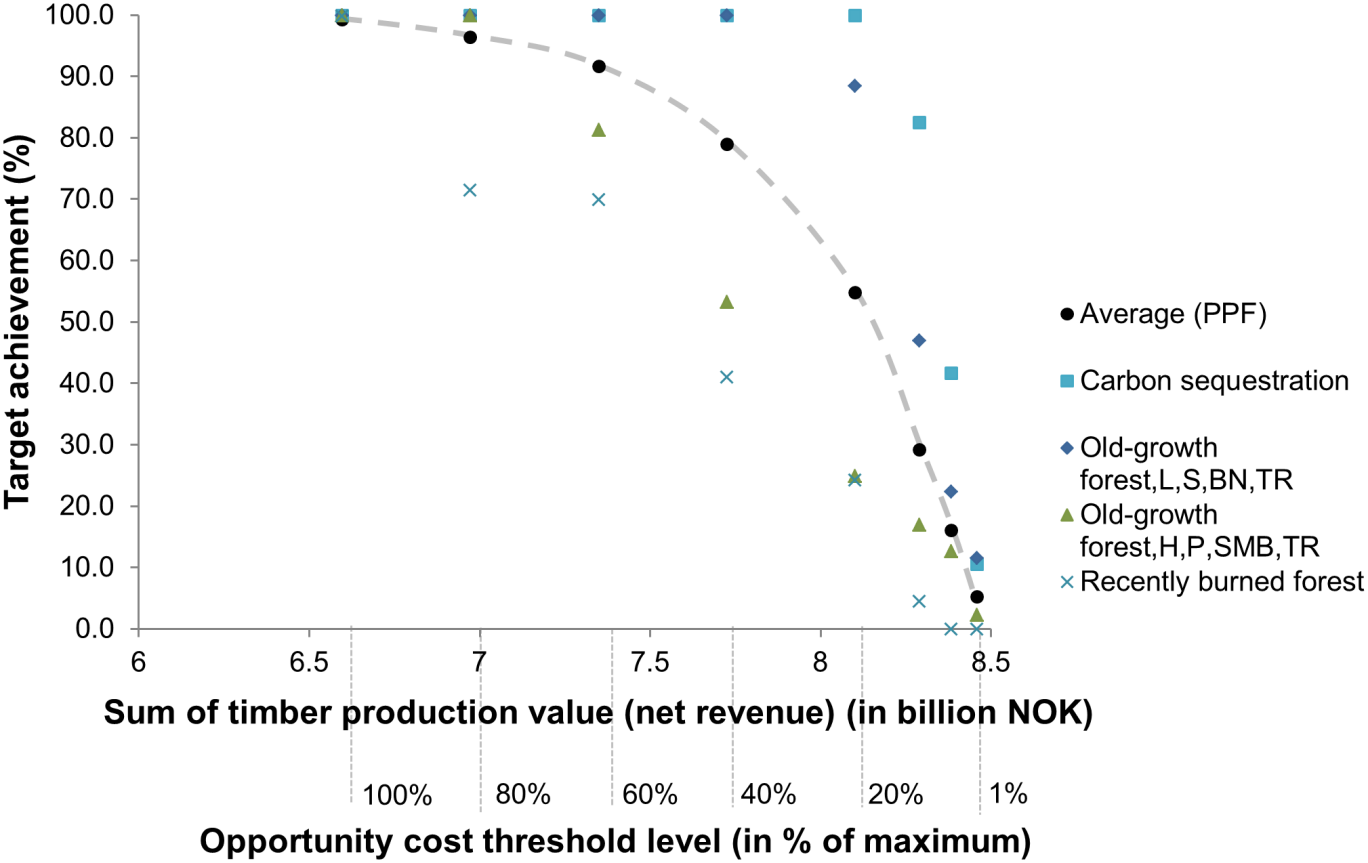
Forest conservation-timber production possibility frontier (PPF) for single, exemplary features. Old-growth forest L, S, BN, TR = impediment and low productivity, spruce dominated, boreonemoral zone, oceanic-inland transition zone. Old-growth forest H, P, SMB, TR = high & very high productivity, pine dominated, South & Mid- boreal zone, oceanic-inland transition zone.

While Figure 3 shows the average target achievement of all 64 features, Figure 4 shows the development of target achievement along changing opportunity cost constraints for single, exemplary features (for all features see Table S3). Some features meet high targets at low (20%) cost constraints (carbon sequestration and one type of low productive old-growth forest), which means that these features did not constrain the solution to a high degree. Some conservation features decreased at higher rates than the average (e.g., one type of high productive forest and recently burned forest). Such features are more costly to be comprehensively conserved in a compact reserve network.

### Distribution of the conservation burden of cost-effective conservation areas

The creation of the policyscape for conservation of biodiversity and ES formed the basis for determining the ‘conservation burden’ across municipalities of Telemark (Table 3, spatial distribution in Figure S2). Conservation burdens across municipalities were slightly shifted in a (hypothetical) scenario with no cost constraint in which approximately 100% of the average target could be achieved compared to the current situation. For instance, while Porsgrunn ranked 6^th^ in terms of the conservation burden of the current policyscape, it ranked 1^st^ in the policyscape of with no cost constraints. The Spearman’s correlation coefficient between the current situation and the scenario with unconstrained costs was r = 0.67. The Spearman’s correlation coefficient between a 60% cost constraint and the unconstrained scenario was r = 0.46, which means that spatial priorities for conservation, and thus conservation burdens, shift with the level of the opportunity cost constraint.

**Table 3:** Absolute and relative conservation burden per municipality in the current situation, with a cost constraint of 60% and with no cost constraint.

		Total opportunity costs^1^ (million NOK)			Relative opportunity costs (NOK per km^2^ forest area)					Ranks relative opportunity costs (NOK/km^2^) (largest to smallest)			
Municipality	Forest areain planningunits (km^2^)	Current	60% costcon-straint	No costcon-straint	Current	60% costcon-straint	No costcon-straint	Totaladdi-tionalburden^2^(million NOK)	Relativeadditionalburden^2^(NOK/km^2^)	Current	60% costcon-straint	No costcon-straint	Addi-tionalburden
**Porsgrunn**	175.5	3.2	30.0	60.0	18,457	170,677	341,874	56.8	323,417	6	4	1	1
**Bamble**	318.8	13.4	110.9	105.0	42,011	347,859	329,518	91.6	287,507	3	3	2	3
**Notodden**	818.8	14.7	39.0	254.3	17,945	47,655	310,558	239.6	292,613	7	15	3	2
**Sauherad**	316.5	13.1	52.3	95.3	41,404	165,259	301,208	82.2	259,804	4	5	4	4
**Kragerø**	341.8	5.8	15.3	88.4	16,979	44,866	258,777	82.6	241,797	8	16	5	5
**Nome**	412.8	54.2	150.9	105.7	131,320	365,660	256,155	51.5	124,835	1	2	6	10
**Drangedal**	1050.8	26.2	63.4	265.6	24,970	60,353	252,817	239.4	227,846	5	12	7	7
**Bø**	239.3	1.5	25.3	56.8	6,122	105,791	237,347	55.3	231,225	14	6	8	6
**Skien**	582.5	7.0	54.8	138.1	11,996	94,157	237,166	131.2	225,169	11	7	9	8
**Siljan**	130.5	9.7	57.6	22.2	74,231	441,457	169,989	12.5	95,758	2	1	10	13
**Nissedal**	855.3	12.5	57.5	110.6	14,630	67,191	129,361	98.1	114,731	9	11	11	11
**Tokke**	712.0	0.4	57.5	89.6	527	80,752	125,781	89.2	125,255	18	9	12	9
**Kviteseid**	662.8	0.9	56.6	72.7	1,433	85,374	109,691	71.7	108,258	17	8	13	12
**Tinn**	880.0	10.4	44.2	91.9	11852	50,223	104,416	81.5	92,564	12	14	14	15
**Fyresdal**	1147.5	5.0	31.2	113.7	4336	27,190	99,098	108.7	94,762	15	18	15	14
**Hjartdal**	649.8	5.6	36.2	57.0	8584	55,755	87,702	51.4	79,118	13	13	16	16
**Seljord**	577.3	1.4	25.2	47.0	2442	43,680	81,465	45.6	79,023	16	17	17	17
**Vinje**	939.8	12.2	64.4	68.9	13025	68,492	73,306	56.6	60,281	10	10	18	18

1 Calculated as foregone net stumpage value.

2 Calculated as the difference between opportunity costs for the case of no cost constraint and the current opportunity costs.

## Discussion

### A policyscape for conservation of biodiversity and ES

The use of spatial planning tools that simultaneously consider conservation of biodiversity and ES in a cost-effective way is a fairly new approach, facilitated by recent advancement in computational science. This approach provides a range of opportunities [8], [10], but still presents challenges in operationalization. Considering ES within biodiversity conservation could be beneficial for incorporating sustainable use of ecosystems [4] when achieving overall conservation goals in land use planning (land sharing), compared to a land use strategy that separates conservation and provision of ES (land sparing). A land sharing principle was included in our study in the partial use zone, which partly allows for the development of synergies between ES, biodiversity and timber production and which complements strict protection zones in policyscapes analysed in this study. In our analysis, we had to rely on expert-backed assumptions when describing the effects of the partial use zone on conservation. This is due to inconclusive knowledge on how restricted logging affects particular elements of biodiversity and ES [17]–[21], [55]. Our study suggests that in forest areas of Telemark the configuration of a policyscape for conservation changes when ES were incorporated (scenario 2) compared to considering only biodiversity conservation criteria (scenario 1). This change was twofold and included a change in total areas assigned to the two protection zones and a change in the spatial configuration of selected sites. Including ES resulted in an increase in the size of the reserve network, a result that is in line with previous studies [8], [36] in that when optimizing for cost-effective representation of conservation targets more areas with lower opportunity costs that contribute to target achievements of both biodiversity and ES are selected.

In contrast to former studies, we used different levels of protection, which enabled us to also specify the change in the policyscape in terms of the spatial distribution of the different zones. Including ES resulted in a strong increase in partial use areas (+36.2%), which was partly expected due to the fact that ES features were considered to be protected for a relatively larger proportion in partial use zones than biodiversity features (Table 1). The difference in spatial configurations of the policyscapes of the two scenarios can partly be explained by relatively low degrees of pairwise spatial overlaps between some ES and the biodiversity features (File S4). It also depends, for instance, on various combinations of biodiversity and ES features on cost-effective sites and proximity of suitable combinations to existing reserves. The difference in spatial configuration leads to different spatial prioritisations of sites to preserve in both zones and thus would have important implications for regional and local decision making.

### Trade-off between commercial timber production and conservation of biodiversity and ES

Including ES next to biodiversity into a conservation scenario reflects different values [4], [59] and as such could lead to more informed policy decisions. In our conservation scenario we thus treated ES of public interest representing partly intangible values (regulating and cultural services) as conservation features with an own target. While in the ES discourse, ES are often treated as generally beneficial [4], here we shed light on potential specific trade-offs among ES and between ES and biodiversity conservation priorities. We included timber production in our analysis, a provisioning service that contributes to private economic benefits, and assessed the form of the trade-off curve (PPF) between timber production on the one hand and cultural and regulating services and biodiversity on the other. The existence of a trade-off on a system level was expected based on our assumption that outside the two conservation zones, elements of biodiversity and ES would not be conserved. This assumption might seem strong, but can be defended by the fact that the dominant form of forest management in Norway is characterised by large-scale clear-cutting [60].

From the PPF, we derive two broad policy conclusions. First, the currently designated nature reserves and landscape protection areas achieved a very low proportion (9.1%) of the conservation targets we set in our scenario. This is partly because the conservation network has not been initially designed to meet the conservation targets we defined in our study. For instance, while attention has been given to rare and threatened forest types [31], we did not assign different conservation targets to the different old-growth forest types, which might in practice be of different importance for forest biodiversity conservation. The result is, however, in agreement with the relatively little forest area that is currently allocated to conservation [31] due to low conservation budgets and conflicts. Further, our findings support the observation of a biased representation of protected areas towards high altitudes and lower opportunity cost areas [61]. This pattern, as well as the under-representation of productive forest in the current conservation network, have also been found for Norway [29], [31], [62]. Our present scenario was deliberately designed to include high productive forest, which partly explains the low target achievement of the current conservation network.

Second, the PPF analysis also provides insights for policy-makers regarding balancing private and public interests. It is a societal choice to determine the level of production of either timber or biodiversity and regulating and cultural ES. The PPF illustrates the high importance given to timber production at present. At the same time, it shows that the relationship between gains in conservation and opportunity costs is not linear. This means that high marginal improvements in conservation can be obtained with relatively smaller increases in costs when a low opportunity cost constraint is relaxed. Thus, with relatively little investment, e.g. spending 40% of the maximum opportunity costs, on average 79% of the scenario targets could be achieved under the assumptions applied in this study. However, inspection of the PPF curve also reveals that lowering the cost constraint reduces the probability of achieving conservation targets for certain habitats (e.g. recently burned forests, high productive forests) within the reserve network. In contrast, carbon sequestration reaches high proportions of the target at low cost which indicates that carbon sequestration can be seen as a co-benefit of protecting biodiversity and other ES, assessed at the scale of all prioritised full and partial protection areas across the study area. This is the inverse logic of the current international debate (i.e. REDD+), where carbon sequestration is targeted to be protected while (unmeasured) biodiversity is a (hoped for) co-benefit [63], but is in agreement with findings of process-based models in recent studies [17].

### Uncertainties in creating the conservation scenario

We encountered several challenges in creating the conservation scenario. The choice of conservation features is a crucial factor that determines the outcome of the site prioritisation. Operationalizing biodiversity conservation requires quantifiable and obtainable indicators [64], [65]. Given restrictions on data availability, we believe that our choice of biodiversity surrogates represents a first step for planning the maintenance of biodiversity in Norwegian forest ecosystems.

Despite the “inevitable subjectivity” in setting conservation targets [66], there is some experience in setting targets for biodiversity conservation [65], [67]. However, setting explicit targets for ES when determining spatial priorities has seldom been done [68]. Current studies using Marxan for ES conservation have pointed out the need for experimentation, explicitly stated assumptions and expertise in setting targets given the absence of this information [8], [11], [36], [37], particularly because ES targets influence the size of the reserve network [35]. A systematic sensitivity test of target levels was, however, out of scope of this current study. ES targets may vary considerably because alternative means are available for substituting forest ES depending on location. Preferences for recreational hiking can shift outside the forest towards mountainous areas. In some areas, feasible technical substitutes for snow slide prevention by forests are available. Since different interests and values are reflected in ES, a systematic stakeholder involvement could provide more insight on target levels for each conservation feature. In a future study, sensitivity analyses could be run based on integrated consultation of forest owners. Because Marxan is a regional level policy-support tool its suitability to be used for conservation planning at the property level is restricted. For example, once priority areas have been identified in a regional planning exercise, local authorities in collaboration with the local forest association try to reach agreement with several adjacent property owners [32]. The conservation outcome is the result of multiple negotiations to achieve a single voluntary nature reserve, the final spatial configuration of which does not depend on the result of a near-optimal site prioritisation software. However, Marxan with Zones could be run iteratively on different agreement configurations to show how marginal conservation burden and target achievement are shifted to other locations, for instance when particular forest owners have declined to agree with an area which would in the first place have been prioritised. Scenario analyses in Marxan with Zones could help planners evaluate the cost-effectiveness of local level conservation decisions, in light of the portfolio of other options, instead of negotiating about one or a few sites at a time.

Another uncertainty in conservation planning lies in the underlying opportunity costs [69]. While we did not test this uncertainty in our analysis, we point out that the advent of forest harvesting for bioenergy could be a ‘game changer’ as it would probably change expected returns to forestry and thus change the spatial distribution of opportunity costs.

Partial use areas, where extractive resource exploitation is restricted, can host high levels of biodiversity [17], [18], [26], [55] and integrating such areas in conservation networks may improve overall conservation effectiveness by reducing costs and conflicts between different economic activities [53]. A combination of non-use and partial-use areas may also help to maintain a landscape that enables processes such as colonization and forest succession, particularly if non-use areas are small. The determination of effectiveness of zones to achieve a conservation target has been identified as a major challenge for conservation planning given limited availability of knowledge [34], [70]. For the sake of simplicity, we assumed a 100% effectiveness to protect biodiversity and ES for the non-use zone, given that this is the highest level of protection that can be achieved. We acknowledge, however, that considering a lower effectiveness level would most probably have led to a larger network of protected areas. In face of natural dynamics and disturbances, effectiveness of conservation areas should be monitored in terms of representativeness and persistence [66], [71]. Because of the uncertainty about the probability of biodiversity persistence in the partial use zone, we explored the consequences of changing the zone contribution for the partial use zone as input in Marxan for 46 biodiversity features (Figure A1 in File S1). With a lower zone contribution, Marxan with Zones tended to select more planning units in the non-use and less in the partial use zone despite considerably lower opportunity costs of the partial use zone; a result that is in line with the findings by Makino et al. [53] in a study of partial protection zones in a marine environment in Fiji.

### Assessing regional level implications of site prioritisation for ES and biodiversity: conservation burden

Decision-making about cost-effective area allocation to protect biodiversity and ES takes place at various levels of governance that may justify the design of new policy instruments. Cost-effective selection of priority sites for conservation can guide measures directed to land owners, for instance by consultation with land owners of selected priority sites on whether they would agree to convert forestry land into voluntary nature reserves, as is the current practice in Norway [32]. While land owners voluntarily entering conservation agreements in Norway are generally compensated for their private opportunity cost [32] accumulated loss of forestry activity in a region may, on the one hand, result in unequal public conservation burdens, particularly across different municipalities. Large protected areas may lead to foregone business opportunities, loss of tax income and additional expenses for municipal governments. On the other hand, protected areas can also provide positive externalities to others, through tourism opportunities and protection of biodiversity more generally. Local governments can be compensated for costs of conservation by state-to-municipal “ecological fiscal transfers” [40], an instrument that has been implemented in Brazil and Portugal, and is currently being considered in several European countries [72]. Ecological fiscal transfers have mainly been based on compensation scaled by area. Proposals to scale ecological fiscal transfers using criteria reflecting the effectiveness of conservation in a municipality have generally been limited by the availability of spatially representative data on biodiversity. We have demonstrated how the creation of cost-effective policyscapes could be used to determine distributional effects of additional conservation efforts.

## Conclusions

Marxan with Zones provides a spatially explicit way to include different types of ES and biodiversity conservation criteria to study a policyscape for cost-effective conservation. We have shown that, in the case of Telemark, including a number of ES shifts priority sites for conservation and increases the area of both a partial use and a non-use zone, compared to a situation where only biodiversity conservation criteria are considered. Conservation of a number of regulating and cultural ES leads to additional conservation efforts, in terms of higher opportunity costs and a larger area protected. We show how carbon sequestration can be viewed as a side-benefit of the protection of other ES and biodiversity in the context of the current Kyoto-based setting of national targets. This is opposite to current thinking about biodiversity as a hoped-for side-benefit of climate mitigation measures under REDD+. The current conservation situation in Telemark clearly prioritises timber production against the protection of biodiversity and ES, and relatively large marginal increases in conservation target achievement could be reached with modest additional investments in terms of compensation for foregone timber production. Our analysis also shows potential differences in conservation burden among municipalities in Telemark, opening the debate on policy instruments such as ecological fiscal transfers that support county-level cost-effective conservation through stimulation of local conservation efforts.

Although the integration of partial use areas into conservation could provide opportunities to increase cost-effectiveness in conservation, significant work is needed to document effectiveness of different levels of protection on particular conservation features. Despite the high level of uncertainty, a policy mix of conservation measures appears to have the potential to contribute to address the complexity of cost-effective conservation problems.

Conservation targets for many aspects of biodiversity and especially ES are currently absent. Conservation planning could be better operationalised with more knowledge on stakeholder preferences about the importance of ES as well as with more ecological knowledge on area size needed to preserve a biodiversity feature.

Our analysis should not be understood as a concrete regional management plan, but rather as an exploratory analysis to provide insights about the current forest conservation situation, about which conservation outcomes could be achieved at which opportunity costs levels. In practice, selection of protected areas is often based on other criteria and motives than cost-effective, comprehensive site prioritisation [61]. Decision makers could use the results of this study to encourage disproportional conservation efforts at local level that achieve cost-effective, near optimal solutions to a conservation problem of multiple biodiversity and ES features. For this to happen, decision makers have to decide to what extent additional information, such as mapping of ES, could be integrated into land-use planning [73]. We have shown how ES mapping, conservation benchmarking and distributional impact analysis using conservation planning tools could inform decision-making and support compensation of land owners’ and local governments’ conservation efforts.

## Supporting Information

Figure S1 **Selection frequency maps.**
(DOC)

Figure S2 **Spatial distribution of the conservation burden.**
(DOC)

Table S1 **Parameter settings of Marxan with Zones.**
(DOC)

Table S2 **Parameters and results of the PPF analysis.**
(DOC)

Table S3 **Target achievement of conservation features.**
(DOC)

File S1 **Sensitivity analysis of the partial use zone contribution.**
(DOC)

File S2 **Marxan with Zones input files – scenario 1.**
(ZIP)

File S3 **Marxan with Zones input files – scenario 2.**
(ZIP)

File S4 **Pairwise spatial overlap of conservation features.**
(XLSX)

Text S1 **Detailed methods.**
(DOC)
