# Supplementary material for: Ecosystem Services and Opportunity Costs Shift Spatial Priorities for Conserving Forest Biodiversity
Source: PLoS One. 2014 Nov 13;9(11):e112557. doi: 10.1371/journal.pone.0112557 (PMC4230974; doi:10.1371/journal.pone.0112557)
Supplement: Figure S1 — Selection frequency maps. (DOC) [file pone.0112557.s001.doc]

**Figure S1**: Selection frequency maps

| 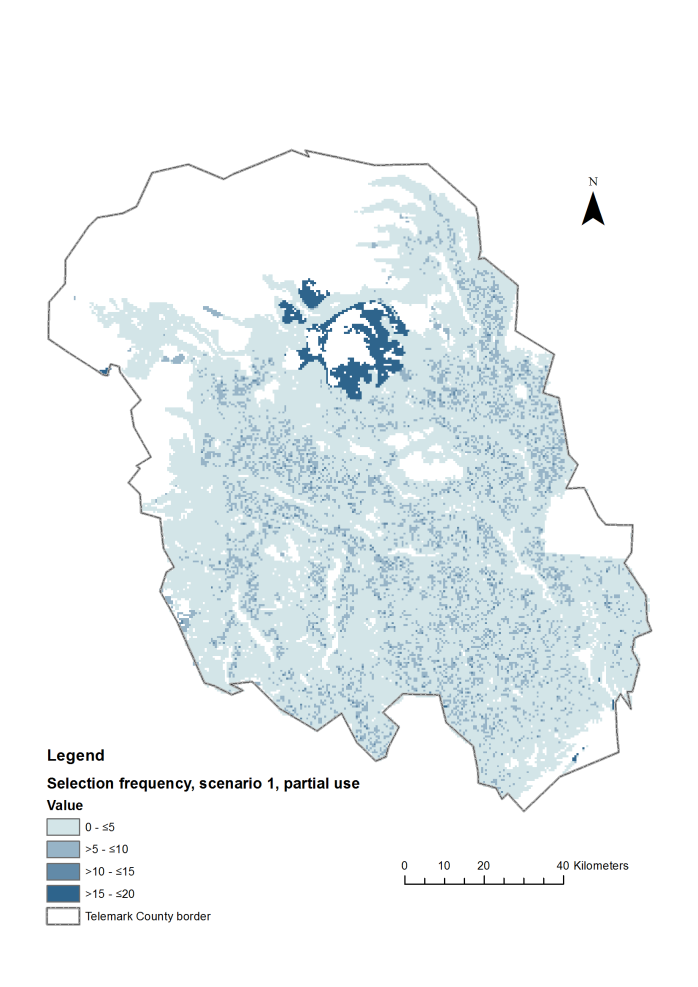 | 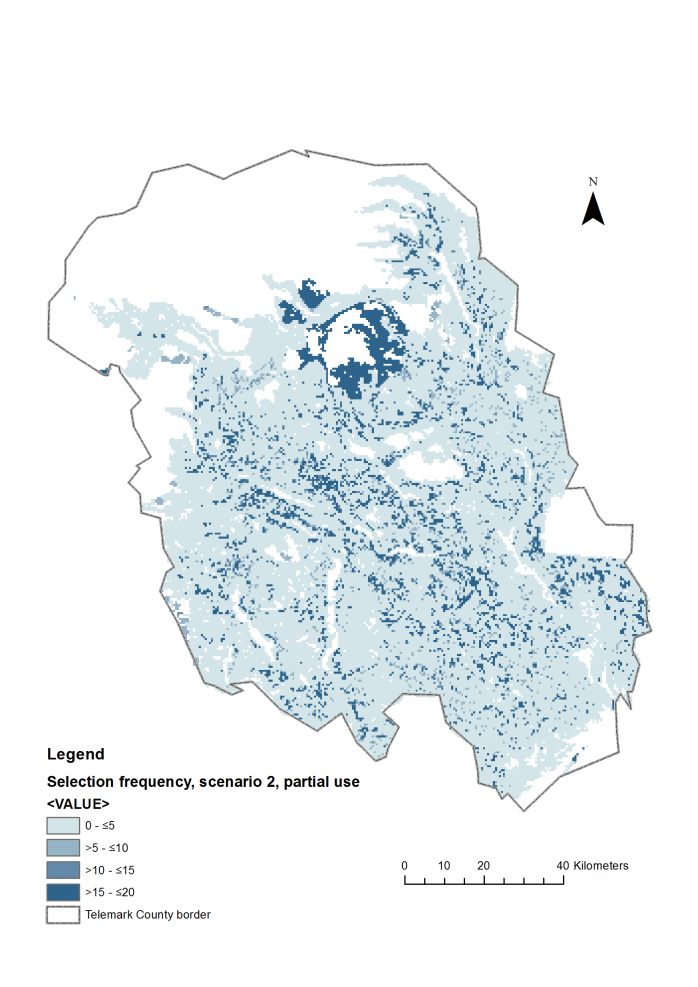 | |
| --- | --- | --- |
| 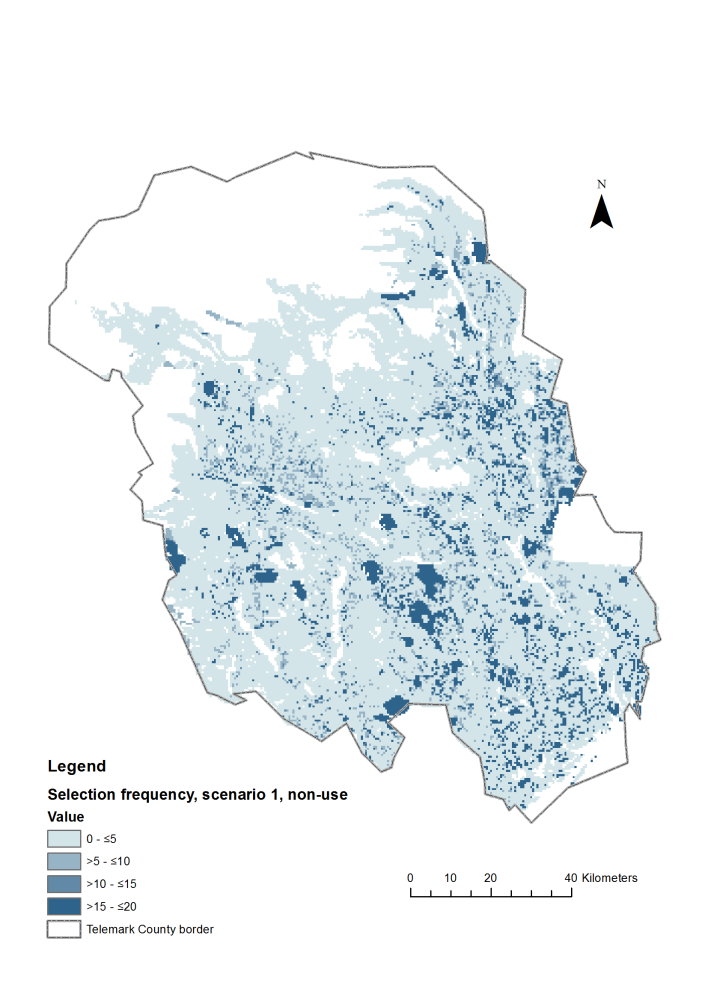 | | 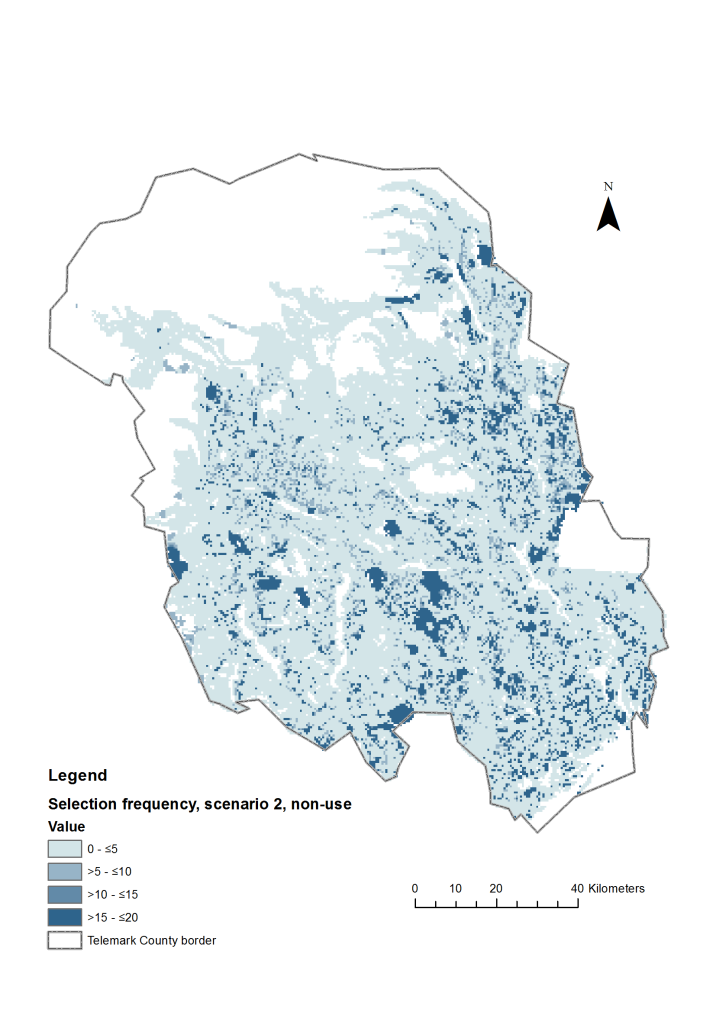 |

Figure S1: Selection frequency per scenario, without cost threshold (left: scenario 1, right: scenario 2, top row: partial use, bottom row: non-use)
