# Supplementary material for: Ecosystem Services and Opportunity Costs Shift Spatial Priorities for Conserving Forest Biodiversity
Source: PLoS One. 2014 Nov 13;9(11):e112557. doi: 10.1371/journal.pone.0112557 (PMC4230974; doi:10.1371/journal.pone.0112557)
Supplement: Figure S2 — Spatial distribution of the conservation burden. (DOC) [file pone.0112557.s002.doc]

**Figure S2: Spatial distribution of the conservation burden**


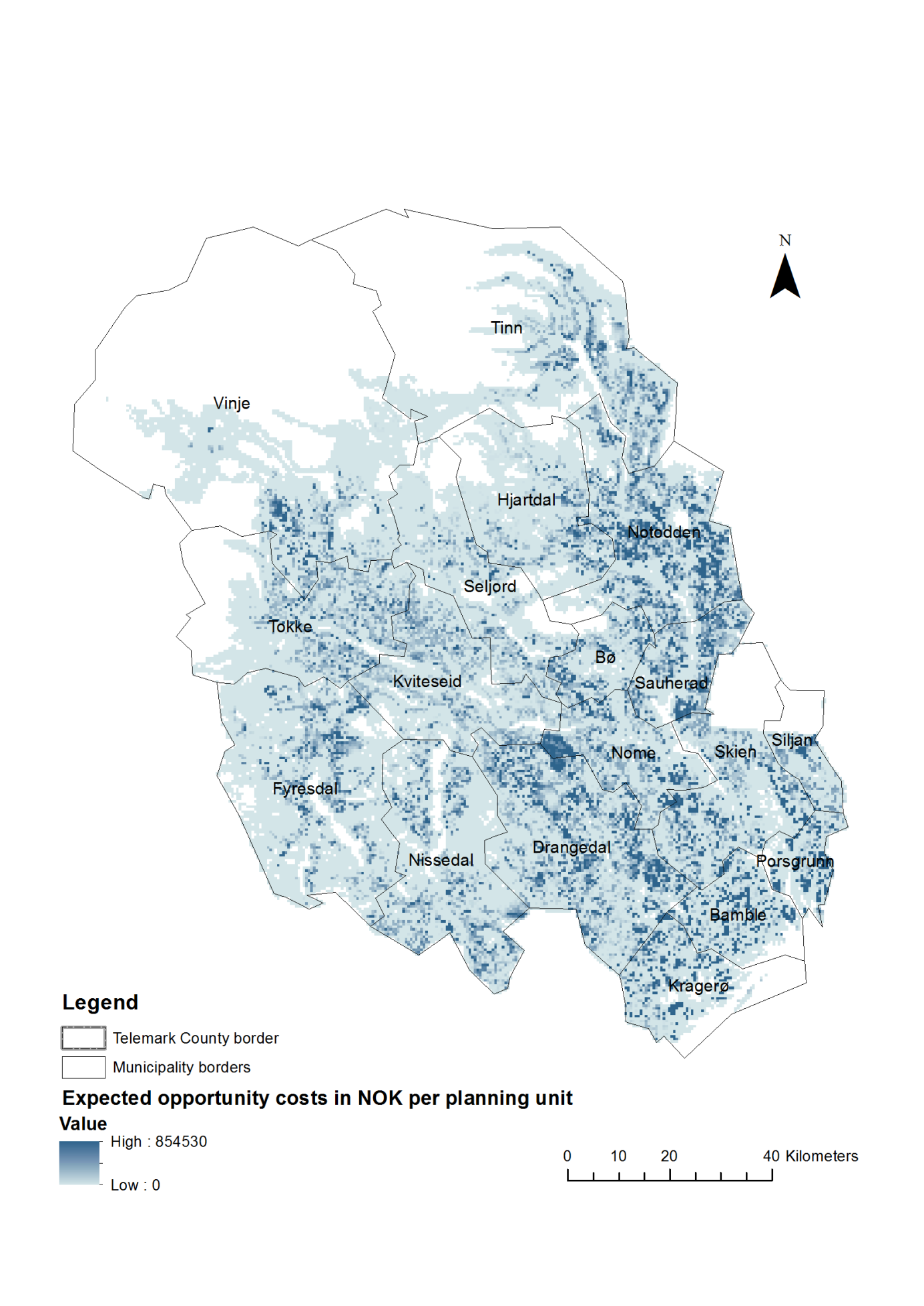


Fig. S2: Spatial distribution of the conservation burden across municipalities of Telemark.
