# Supplementary material for: Ecosystem Services and Opportunity Costs Shift Spatial Priorities for Conserving Forest Biodiversity
Source: PLoS One. 2014 Nov 13;9(11):e112557. doi: 10.1371/journal.pone.0112557 (PMC4230974; doi:10.1371/journal.pone.0112557)
Supplement: Table S1 — Parameter settings of Marxan with Zones. (DOC) [file pone.0112557.s003.doc]

**Table S1: Parameter settings of Marxan with Zones**

Table S1: MARXAN input file and parameters. For abbreviations see .

| scenario 1 | scenario 2 |
| --- | --- |
| General Parameters  BLM 50  PROP 0.5  RANDSEED -1  NUMREPS 20  Annealing Parameters  NUMITNS 1000000  STARTTEMP -1  NUMTEMP 10000  Cost Threshold  COSTTHRESH 0  THRESHPEN1 14.0  THRESHPEN2 1.0  Program control.  RUNMODE 1  MISSLEVEL 1  ITIMPTYPE 0  HEURTYPE -1  CLUMPTYPE 0  VERBOSITY 3 | General Parameters  BLM 50  PROP 0.5  RANDSEED -1  NUMREPS 20  Annealing Parameters  NUMITNS 1000000  STARTTEMP -1  NUMTEMP 10000  Cost Threshold  COSTTHRESH 0  THRESHPEN1 14.0  THRESHPEN2 1.0  Program control.  RUNMODE 1  MISSLEVEL 1  ITIMPTYPE 0  HEURTYPE -1  CLUMPTYPE 0  VERBOSITY 3 |
| Feature penalty factor (FPF): 0.5 for all features | Feature penalty factor: 0.5 for all features, except for feature snow slide prevention FPF=1 |

**Reference**

1. Watts ME, Klein CJ, Stewart R, Ball IR and Possingham HP (2008) Marxan with Zones (v1.0.1): Conservation Zoning using Spatially Explicit Annealing, a Manual.
