# Supplementary material for: Ecosystem Services and Opportunity Costs Shift Spatial Priorities for Conserving Forest Biodiversity
Source: PLoS One. 2014 Nov 13;9(11):e112557. doi: 10.1371/journal.pone.0112557 (PMC4230974; doi:10.1371/journal.pone.0112557)
Supplement: Table S2 — Parameters and results of the PPF analysis. (DOC) [file pone.0112557.s004.doc]

**Table S2: Parameters and results of the PPF analysis**

Table S2. Parameters and results of the PPF analysis

| Cost constraint level | Cost of the best solution (NOK) | Timber production (NOK) | Average target achievement | Feature penalty factor | Cost threshold penalty factor 1 |
| --- | --- | --- | --- | --- | --- |
| 100 | 1,881,837,140 | 6,595,649,722 | 99.3 | 1.2 | 14 |
| 80 | 1,505,378,254 | 6,972,108,608 | 96.4 | 6 | 14 |
| 60 | 1,129,170,896 | 7,348,315,966 | 91.6 | 6 | 14 |
| 40 | 752,769,819 | 7,724,717,043 | 79.0 | 4 | 14 |
| 20 | 376,367,471 | 8,101,119,392 | 54.8 | 2 | 14 |
| 10 | 188,183,856 | 8,289,303,007 | 29.2 | 1 | 14 |
| 5 | 94,091,852 | 8,383,395,010 | 16.1 | 0.5 | 14 |
| 1 | 18,823,596 | 8,458,663,266 | 5.2 | 0.5 | 210 |
