# Supplementary material for: Ecosystem Services and Opportunity Costs Shift Spatial Priorities for Conserving Forest Biodiversity
Source: PLoS One. 2014 Nov 13;9(11):e112557. doi: 10.1371/journal.pone.0112557 (PMC4230974; doi:10.1371/journal.pone.0112557)
Supplement: Table S3 — Target achievement of conservation features. (DOC) [file pone.0112557.s005.doc]

**Table S3: Target achievement of conservation features**

Table S3: Target achievement of conservation features with different opportunity cost thresholds.

|  | **Cost constraint (%)** | **100** | **80** | **60** | **40** | **20** | **10** | **5** | **1** |
| --- | --- | --- | --- | --- | --- | --- | --- | --- | --- |
| **No.** | **Feature name** | **Target achievement (%)** | | | | | | | |
| **1** | Existence of wilderness-like areas | 98.3 | 86.6 | 78.9 | 71.2 | 46.4 | 21.8 | 3.5 | 0.1 |
| **2** | Recreational hiking | 100.0 | 100.0 | 100.0 | 100.0 | 100.0 | 72.5 | 51.7 | 9.2 |
| **3** | Carbon storage | 100.0 | 100.0 | 100.0 | 100.0 | 76.4 | 40.6 | 20.8 | 5.2 |
| **4** | Carbon sequestration | 100.0 | 100.0 | 100.0 | 100.0 | 100.0 | 82.6 | 41.7 | 10.5 |
| **5** | Snow slide prevention | 99.5 | 95.5 | 93.4 | 89.9 | 80.0 | 65.0 | 43.7 | 15.8 |
| **6** | Old-growth forest,L,B,SMB,OC | 100.0 | 100.0 | 100.0 | 99.1 | 65.9 | 35.8 | 13.8 | 11.6 |
| **7** | Old-growth forest,L,B,SMB,TR | 100.0 | 100.0 | 100.0 | 99.8 | 61.2 | 28.0 | 13.8 | 10.9 |
| **8** | Old-growth forest,L,B,BN,OC | 100.0 | 100.0 | 100.0 | 87.5 | 71.9 | 31.3 | 9.4 | 6.3 |
| **9** | Old-growth forest,L,B,BN,TR | 100.0 | 100.0 | 90.9 | 66.8 | 38.8 | 21.8 | 13.9 | 2.0 |
| **10** | Old-growth forest,L,M,SMB,OC | 100.0 | 100.0 | 100.0 | 97.7 | 46.3 | 19.2 | 12.4 | 5.6 |
| **11** | Old-growth forest,L,M,SMB,TR | 100.0 | 100.0 | 100.0 | 100.0 | 63.9 | 28.4 | 14.2 | 10.8 |
| **12** | Old-growth forest,L,M,BN,OC | 100.0 | 100.0 | 100.0 | 100.0 | 100.0 | 0.0 | 0.0 | 0.0 |
| **13** | Old-growth forest,L,M,BN,TR | 100.0 | 100.0 | 100.0 | 100.0 | 70.1 | 47.0 | 12.0 | 11.1 |
| **14** | Old-growth forest,L,P,SMB,OC | 100.0 | 100.0 | 95.2 | 66.0 | 39.0 | 21.7 | 12.0 | 5.6 |
| **15** | Old-growth forest,L,P,SMB,TR | 100.0 | 100.0 | 84.3 | 57.5 | 27.4 | 17.2 | 13.2 | 2.6 |
| **16** | Old-growth forest,L,P,BN,OC | 100.0 | 100.0 | 96.8 | 70.3 | 41.9 | 21.7 | 12.5 | 1.6 |
| **17** | Old-growth forest,L,P,BN,TR | 100.0 | 99.9 | 82.6 | 54.9 | 30.9 | 19.0 | 14.1 | 3.5 |
| **18** | Old-growth forest,L,S,SMB,OC | 100.0 | 99.7 | 90.8 | 61.6 | 25.4 | 14.4 | 13.3 | 5.2 |
| **19** | Old-growth forest,L,S,SMB,TR | 100.0 | 100.0 | 100.0 | 87.3 | 50.0 | 24.3 | 14.5 | 7.6 |
| **20** | Old-growth forest,L,S,BN,OC | 100.0 | 100.0 | 100.0 | 93.9 | 83.3 | 39.4 | 33.3 | 6.1 |
| **21** | Old-growth forest,L,S,BN,TR | 100.0 | 100.0 | 100.0 | 100.0 | 88.4 | 47.0 | 22.4 | 11.6 |
| **22** | Old-growth forest,L,C,SMB,OC | 100.0 | 100.0 | 99.7 | 80.3 | 37.9 | 20.3 | 14.0 | 7.3 |
| **23** | Old-growth forest,L,C,SMB,TR | 100.0 | 100.0 | 100.0 | 92.7 | 49.1 | 24.3 | 13.9 | 8.4 |
| **24** | Old-growth forest,L,C,BN,OC | 100.0 | 100.0 | 100.0 | 98.8 | 86.4 | 35.8 | 29.6 | 12.3 |
| **25** | Old-growth forest,L,C,BN,TR | 100.0 | 100.0 | 100.0 | 94.8 | 62.2 | 29.5 | 17.1 | 9.0 |
| **26** | Old-growth forest,H,B,SMB,OC | 100.0 | 100.0 | 100.0 | 100.0 | 87.7 | 61.5 | 49.2 | 18.5 |
| **27** | Old-growth forest,H,B,SMB,TR | 100.0 | 99.9 | 95.2 | 74.4 | 46.0 | 23.5 | 13.3 | 5.7 |
| **28** | Old-growth forest,H,B,BN,OC | 100.0 | 100.0 | 100.0 | 77.8 | 69.4 | 16.7 | 8.3 | 0.0 |
| **29** | Old-growth forest,H,B,BN,TR | 100.0 | 99.0 | 75.5 | 53.4 | 29.2 | 18.3 | 11.6 | 1.2 |
| **30** | Old-growth forest,H,M,SMB,OC | 100.0 | 100.0 | 100.0 | 96.8 | 54.8 | 29.0 | 9.7 | 9.7 |
| **31** | Old-growth forest,H,M,SMB,TR | 100.0 | 100.0 | 99.8 | 84.3 | 47.4 | 23.8 | 13.1 | 7.8 |
| **32** | Old-growth forest,H,M,BN,OC | 100.0 | 100.0 | 100.0 | 92.3 | 61.5 | 7.7 | 0.0 | 0.0 |
| **33** | Old-growth forest,H,M,BN,TR | 100.0 | 98.7 | 87.8 | 64.7 | 43.6 | 26.3 | 20.5 | 9.0 |
| **34** | Old-growth forest,H,P,SMB,OC | 100.0 | 99.4 | 99.7 | 71.6 | 36.3 | 23.5 | 11.9 | 8.2 |
| **35** | Old-growth forest,H,P,SMB,TR | 100.0 | 100.0 | 81.3 | 53.2 | 24.9 | 16.9 | 12.7 | 2.3 |
| **36** | Old-growth forest,H,P,BN,OC | 100.0 | 100.0 | 98.2 | 73.0 | 36.0 | 15.1 | 11.3 | 2.3 |
| **37** | Old-growth forest,H,P,BN,TR | 100.0 | 99.9 | 76.3 | 48.4 | 26.8 | 17.7 | 14.2 | 2.9 |
| **38** | Old-growth forest,H,S,SMB,OC | 100.0 | 99.5 | 84.7 | 63.5 | 35.4 | 26.3 | 19.4 | 8.2 |
| **39** | Old-growth forest,H,S,SMB,TR | 100.0 | 100.0 | 96.3 | 75.4 | 41.9 | 22.1 | 13.1 | 6.4 |
| **40** | Old-growth forest,H,S,BN,OC | 100.0 | 100.0 | 97.2 | 81.5 | 62.0 | 43.5 | 25.0 | 3.7 |
| **41** | Old-growth forest,H,S,BN,TR | 100.0 | 100.0 | 97.2 | 87.7 | 65.6 | 32.4 | 18.3 | 6.0 |
| **42** | Old-growth forest,H,C,SMB,OC | 100.0 | 100.0 | 99.2 | 90.9 | 46.4 | 27.9 | 20.8 | 10.2 |
| **43** | Old-growth forest,H,C,SMB,TR | 100.0 | 100.0 | 99.3 | 75.6 | 40.0 | 23.1 | 13.2 | 6.3 |
| **44** | Old-growth forest,H,C,BN,OC | 100.0 | 100.0 | 100.0 | 82.9 | 67.5 | 35.9 | 25.6 | 7.7 |
| **45** | Old-growth forest,H,C,BN,TR | 100.0 | 99.7 | 84.1 | 65.5 | 38.3 | 18.3 | 16.8 | 6.4 |
| **46** | Forest corridor 1 | 100.0 | 100.0 | 100.0 | 60.0 | 20.0 | 20.0 | 20.0 | 0.0 |
| **47** | Forest corridor 2 | 100.0 | 96.6 | 84.7 | 64.4 | 38.0 | 18.8 | 6.0 | 0.5 |
| **48** | Forest corridor 3 | 100.0 | 99.4 | 96.7 | 93.9 | 74.8 | 48.4 | 21.1 | 7.4 |
| **49** | Forest corridor 4 | 100.0 | 97.8 | 89.7 | 80.1 | 39.1 | 26.4 | 12.4 | 1.8 |
| **50** | Forest corridor 5 | 100.0 | 100.0 | 100.0 | 99.4 | 87.7 | 52.9 | 21.6 | 3.2 |
| **51** | Forest corridor 6 | 100.0 | 100.0 | 100.0 | 100.0 | 89.3 | 34.7 | 40.7 | 4.0 |
| **52** | Forest vegetation patches (very important) | 94.5 | 84.9 | 80.5 | 73.0 | 55.5 | 23.2 | 4.3 | 0.0 |
| **53** | Forest vegetation patches (important) | 93.2 | 79.2 | 71.5 | 58.7 | 38.7 | 14.1 | 3.6 | 0.2 |
| **54** | Forest vegetation patches (locally important) | 100.0 | 98.1 | 94.8 | 78.3 | 46.7 | 18.4 | 0.5 | 0.0 |
| **55** | hollow deciduous trees | 96.5 | 79.2 | 71.4 | 59.8 | 39.3 | 22.8 | 3.9 | 0.1 |
| **56** | late successional forests with deciduous trees | 97.5 | 86.3 | 77.7 | 72.2 | 56.3 | 35.0 | 14.6 | 0.0 |
| **57** | logs | 95.2 | 84.3 | 77.8 | 70.0 | 56.1 | 36.7 | 14.9 | 2.8 |
| **58** | old trees | 97.6 | 88.5 | 79.3 | 76.8 | 57.5 | 35.0 | 13.0 | 0.9 |
| **59** | rich ground vegetation | 94.2 | 80.2 | 71.3 | 59.7 | 42.1 | 24.8 | 7.0 | 0.4 |
| **60** | snags | 96.8 | 89.7 | 78.2 | 75.0 | 69.0 | 45.8 | 29.2 | 6.1 |
| **61** | trees with nutrient-rich bark | 98.9 | 93.0 | 89.2 | 80.6 | 68.1 | 48.2 | 18.5 | 3.9 |
| **62** | trees with pendant lichens | 96.4 | 81.6 | 72.4 | 61.0 | 48.7 | 21.8 | 11.5 | 0.0 |
| **63** | recently burned forest | 99.8 | 71.5 | 70.0 | 41.0 | 24.2 | 4.5 | 0.0 | 0.0 |
| **64** | stream gorges | 94.8 | 82.4 | 75.3 | 71.4 | 52.3 | 20.7 | 0.0 | 0.0 |

L: impediment and low productivity, H: medium, high & very high productivity, B: broadleaf forest, M: mixed forest, P:pine forest, S: spruce forest, C: coniferous mixed forest, BN: boreonemoral, SMB: south & middle boreal, TR=transition zone, OC: clear & weak oceanic
