# Supplementary material for: Ecosystem Services and Opportunity Costs Shift Spatial Priorities for Conserving Forest Biodiversity
Source: PLoS One. 2014 Nov 13;9(11):e112557. doi: 10.1371/journal.pone.0112557 (PMC4230974; doi:10.1371/journal.pone.0112557)
Supplement: File S1 — Sensitivity analysis of the partial use zone contribution. (DOC) [file pone.0112557.s006.doc]

**File S1: Sensitivity analysis of the partial use zone contribution**

A critical question about combining strict protection and partial use areas for conservation is to which extent partial use areas can insure the persistence of biodiversity given considerable uncertainty about the role of partial use areas for conservation , which has also been discussed in Norway . We address this question by conducting a sensitivity analysis of how the policyscape changes with varying levels of effectiveness of partial use areas to conserve biodiversity, and discuss what implications this has for the functional roles of strict and partial protection conservation instruments in the landscape. The effect of the probability of persistence in the partial use zone was tested in a sensitivity analysis by changing the value of zone contribution for the 40 forest types and the 6 corridors from 50 to 10% at 10% intervals. This analysis was performed *ceteris paribus* for a cost constraint of 60% of the maximum opportunity costs for scenario 2.

Result

With a lower zone contribution, i.e. lower effectiveness of partial use areas to protect biodiversity in old-growth forest and forest corridors, more area is protected in the non-use zone, while less area is protected in the partial use zone (Figure SX1). Ceteris paribus, average target achievement decreases slightly when a lower zone contribution is set, while total area protected decreases with a slightly higher magnitude.


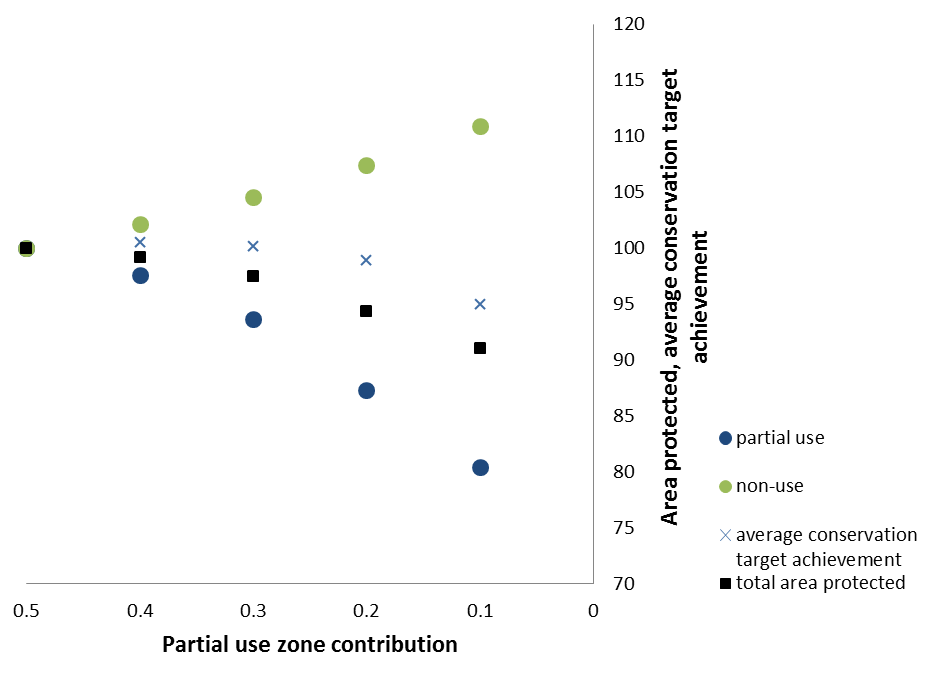


Figure A1: Effect of zone contribution of the partial use zone on total area protected, area protect in the partial use and non-use zone, and average target achievement. Values for a zone contribution of 0.5 were set at 100. This analysis was done on scenario 2 with a cost threshold of 60% of the maximum cost needed to reach 100% target achievement and with current protected areas locked-in.

**References**

1. Faith DP (2012) Common ground for biodiversity and ecosystem services: The "partial protection" challenge [v1; ref status: indexed, http://f1000r.es/QPrmmt]. F1000Research 2012 1.

2. Søgaard G, Eriksen R, Astrup R and Øyen B-H (2012) Effekter av ulike miljøhensyn på tilgjengelig skogareal og volum i norske skoger. Rapport fra Skog og Landskap 2/2012. Ås: Skog og Landskap.

3. Barton DN, Lindhjem H, Rusch GM, Sverdrup-Thygeson A, Blumentrath S, et al. (2012) Assessment of existing and proposed policy instruments for biodiversity conservation in Norway. POLICYMIX report No 1/2012. Oslo: NINA.
