# Supplementary material for: Ecosystem Services and Opportunity Costs Shift Spatial Priorities for Conserving Forest Biodiversity
Source: PLoS One. 2014 Nov 13;9(11):e112557. doi: 10.1371/journal.pone.0112557 (PMC4230974; doi:10.1371/journal.pone.0112557)
Supplement: Text S1 — Detailed methods. (DOC) [file pone.0112557.s010.doc]

**Text S1: Detailed methods**

*Old-growth forest cross tabularisation*

The forest types were distinguished based on a cross tabularization (5 x 2 x 2 x 2). Five forest cover classes (spruce, pine, deciduous, mixed, coniferous mixed) from a remote sensing based forest map were crossed with two vegetation zones representing altitudinal vegetation ranges, and two regional climate zones representing a gradient from coast to inland climate. Two classes indicating the potential for forestry production (impediment & low; medium, high & very high) retrieved from the national land resources dataset were used as a surrogate for site ecological productivity. Old forest was determined as the highest age quartile per forest cover type in the SAT-SKOG forest map.

*Targets*

The target for wilderness-like areas was set at 100% in order to reflect the political goal to protect these areas . The target for recreational hiking was arbitrarily set at 20%. The target for forest carbon sequestration was determined based on an estimation that 3 million tons of carbon dioxide sequestration should be accounted for in Norway according to the country’s obligations to the Kyoto protocol . As Telemark has 7.16% of all forest in Norway, the corresponding amount is 0.215 million t C. This is equivalent to 5.57% of all estimated carbon sequestered in forest in Telemark . The target for carbon storage, while missing a concrete political goal, was tentatively set at 10%. Snow slide prevention was set at 100%, implying that there are no alternative means for risk mitigation in forested slopes exposed to snow slides. The 40 different forest types cover a relatively large area (140.068 ha) and should therefore have a significant effect on the basic shape of the reserve network. We set the target at 50% for each type so that we ensured that the current conservation gap in lowland and productive forests can be closed. For the remaining biodiversity features the targets have been tentatively set in order to reflect their ecological importance. The targets for forest corridors were set at 50%. In the case of priority habitats for conservation, locally important habitats were set at 50%, whereas as the other two classes were set at 100%. Targets for important forest habitats were set at 100% as well (Table 1).

*Definition of zones*

In Norway, non-use areas are nature reserves, with the strongest protection form according to the Nature Diversity Act (Naturmangfoldloven, LOV-2009-06-19-100). We considered the partial use zone as an ‘umbrella’ zone covering three different current forms of protection where forestry is partially restricted, namely landscape protection areas, mountain forest (‘fjellskog’), and outdoor recreation areas (‘friluftsområder’). In landscape protection areas forestry activities are generally allowed, but particular regulations regarding, for instance, the shift of the dominant tree type, the felling of large trees, and harvest cycles could apply on the forest area beyond the ordinary forestry environmental regulations. Mountain forest refers to forest that occurs on sites where the economic profit is less important than the forest’s environmental protection function, such as snow-slide and flood control. Approximately 17% of the productive forest in Norway is regarded as mountain forest according to the Forestry Law (Lov om skogbruk, LOV-2005-05-27-31) . In outdoor recreation areas there are restrictions to forestry due to consideration of forest cabin fields or other forms of outdoor recreation .

*Zone targets*

The ES existence of areas without technical interference was assigned completely to the non-use zone, as this ES is by definition considered to have no or very low use levels. The recreational hiking target was distributed equally among non-use and partial use, considering that that hiking is compatible with restricted forestry activity. Snow slide prevention is conventionally provided by partial use zones . We thereby assigned this feature completely to the partial use zone (100%). Carbon sequestration and storage can be impaired due to soil disturbances and carbon removal in the form of harvested wood. Therefore, these services should primarily be preserved in a non-use zone (75%). Comparably, we considered old-growth forest types to be best protected in non-use areas (75% of the target). For corridors, we tentatively distributed targets equally among the non-use (50%) and partial use (50%) zone. We assigned all priority habitats for conservation completely to the non-use zone.

*Zone contributions*

In light of the uncertainty of effectiveness of the partial use zone to conserve features, we assumed in a first step that the probability of persistence of old-growth forest species and of corridors in the partial protection zone was 50%. For forest habitats of particular conservation importance which are supposed to be subject to high levels of threat , we assumed that the partial use zone is insufficient for protection. In the case of ES, we assumed the partial use zone would have a 50% probability of maintaining recreational hiking values whereas the capacity for carbon sequestration and storage would be reduced in accordance to the levels of use (25% foregone logging in this zone, see below ‘opportunity costs’). Snow slide protection can be fully provided by multi-use areas , and the contribution for this ES was set at 1.0.

*Parameter adjustments*

Marxan with Zones requires a number of parameters to be set. The boundary length modifier (BLM) was set according to methods described in in order to guide the software to select a compact or spatially coherent reserve network. A feature penalty factor, which steers the software to find comprehensive solutions to the optimisation problem, was set in order to reach a high target achievement in each scenario according to the iterative procedure described in . Detailed parameter values of the specific runs are shown in Table S1.

*Opportunity costs*

The approach for calculation of the opportunity costs consisted of five subtasks. First the GAYA-J model is applied to data from national forest inventory (NFI) plots and GIS data is prepared so as to match the characteristics of the field data from NFI. The GAYA-J model accounts for differences in site index, terrain characteristics, forwarding distance to the nearest transportation point, and calibrated cost functions to calculate site specific stumpage values. In the next step the NFI plots with the timber value estimates from GAYA-J are spatially joined with the prepared GIS data at the plot locations. Then Generalised Linear Models (GLM) for opportunity costs were created based on the joined data. Finally, the GLM were applied to the significant map layers with area-wide coverage.

*Expert workshop*

An expert workshop was organised to verify targets, zone targets and assumptions on effectiveness. Participants came from the Norwegian Ministry of Climate and Environment, the Norwegian Environmental Agency, and scientists from the Norwegian Institute for Nature Research. Notably, all participants were from the public sector interests that had been invited. A number of private and NGO forestry, recreation and environmental sector interests were invited but declined to participate.

*Participants list*

David N. Barton, Norwegian Institute for Nature Research, Oslo

Matthias Schröter, Environmental Systems Analysis Group, Wageningen University

Graciela M. Rusch, Norwegian Institute for Nature Research, Trondheim

Stefan Blumentrath, Norwegian Institute for Nature Research, Oslo

Björn Nordén, Norwegian Institute for Nature Research, Oslo

Erik Framstad, Norwegian Insitute for Nature Research, Oslo

Tor Erik Brandrud, Norwegian Insitute for Nature Research, Oslo

Anne Sverdrup-Thygeson, Norwegian Insitute for Nature Research, Oslo

Asbjørn Tingstad, Norwegian Environmental Agency

Øyvind Lone, Norwegian Ministry of Climate and Environment

Ingunn Aanes, Norwegian Ministry of Climate and Environment

*Structure of the workshop*

Participants were presented with the principle of the analysis and with the assumptions made on the input parameters, in particular the selection of conservation features, targets, zone targets and zone contribution of the partial use zone.

The following questions were addressed in group discussions.

1) Have the conservation features in the model been reasonably chosen?  Are there any features missing?

2) Are the conservation targets for each feature reasonably set? Would you suggest any alternative targets from your perspective?

3) Is the distribution of targets among the different zones reasonable, i.e. which fraction should be preserved in the partial use zone and which in the non-use zone (strict protection)?

4) Is the effectiveness of the partial use zone realistic for each feature? Would you suggest other levels of effectiveness for single features?

*Summary of results*

1) Selection of conservation features

Participants confirmed that the collected conservation features were representative in a Norwegian context and covered, given restricted (spatial) data availability, a variety of biodiversity aspects in a good way.

2) Setting conservation targets

This aspect was considered to be challenging, “normative” and abstract by many participants. For the target for existence of wilderness-like areas it was pointed out that the preservation of such areas is contested and depends on political decisions. A recreation target could not be set after discussion as it was perceived as too abstract. The target for carbon storage for Telemark would depend on international agreements that would bind Norway to also protect carbon stored in forest ecosystems and can be tentatively set until more clarity is attained from international climate negotiations. The target for carbon sequestration, which was determined from the Kyoto process on climate negotiation was perceived as reasonable. Setting targets for old-growth forest was perceived as reasonable but challenging. The final outcomes of a reserve network, of which old-growth forest should be a large part, would need to be consistent with the Aichi biodiversity targets stating that reserves to protect terrestrial biodiversity should cover 17% of the area. Participants considered the 50% target as reasonable in case it would lead, together with the protection of other features, to 17% of the areas of Telemark protected. The target for corridors was discussed as follows. The selected corridors are of a rather large size. In case the 50% of the area target would be achieved ‘along’ a connecting line, then the target would be considered sufficient. If, however, a corridor would only be preserved 50% in a way that it would appear to be disconnected, then the target could have been chosen higher. The site selection through Marxan cannot be steered in a way that it choses areas along a connecting line. However, Marxan is set to find connected areas, so that it seemed reasonable to assume that a corridor would be represented sufficiently in a selected reserve. Important forest habitats were considered to be valuable for conservation, so that the 100% target was confirmed.

3) Setting zone targets

Zone targets were verified for wilderness-like areas, carbon storage, carbon sequestration and snow slide prevention. Recreational hiking was perceive as too abstract and could not be verified. Old-growth forest could potentially be preserved 100% in non-use areas. However, participants also discussed that it depends on the concrete design of the management plan for a partial use area, for instance, the creation of shifting succession areas could contribute in combining use and preservation of old-growth structures. The same argumentation holds for corridors. For priority habitats and other important forest habitats it was discussed that they could partially also be preserved in partial use areas. However, for simplicity reasons and given uncertainties around the effectiveness of partial use areas, we did not assign a partial use zone target to forest habitats.

4) Effectiveness of the partial use zone

Discussion was restricted to conservation features for which a part of the feature was also protected in the partial use zone. Effectiveness for recreational hiking was considered suitable to be at 100% as recreational activities are not hampered to any large degree in case logging is partially restricted. Overall enjoyment of hiking should benefit from total logging restriction. However, data on preferences of hikers is scarce and thus a difference between non-use and partial use zones in the effectiveness to provide the ecosystem service recreational hiking is difficult to quantify. Furthermore, as pointed out, recreational forests have been identified as one form of forest where restricted harvest is possible. Carbon storage and sequestration effectiveness was considered reasonable at 25%, which corresponded to harvest levels. For snow slide prevention the effectiveness was accepted to be at 100% as restricted harvest is compatible with the snow slide prevention function. For old-growth forests and corridors it was pointed out that effectiveness is uncertain and depends to a large degree on the actual management plans for an area.

**References**

1. Gjertsen AK and Nilsen J-E (2012) SAT-SKOG: et skogkart basert på tolking av satellittbilder. In: NFLI, editor editors. Rapport fra Skog og Landskap. Ås: National Forest and Landscape Institute (NFLI, Skog og Landskap).

2. Moen A (1999) National Atlas of Norway: Vegetation. Hønefoss: Norwegian Mapping Authority.

3. NFLI (2010) Arealressurskart AR5. Ås: National Forest and Landscape Institute (NFLI, Skog og Landskap).

4. Directorate for Nature Management (1995) Ingrepsfrie naturområder i Norge. Registrert med bakgrunn i avstand fra tyngre tekniske inngrep. DN-rapport. Trondheim: Directorate for Nature Management.

5. Norwegian Ministry for the Environment (2012) Meld. St. 21 (Melding til Stortinget): Norsk klimapolitikk.

6. Schröter M, Barton DN, Remme RP and Hein L (2014) Accounting for capacity and flow of ecosystem services: A conceptual model and a case study for Telemark, Norway. Ecol Indic 36: 539-551.

7. Framstad E, Blindheim T, Erikstad L, Thingstad PG and Sloreid SE (2010) Naturfaglig evaluering av norske verneområder. NINA rapport 535. Oslo: NINA.

8. Søgaard G, Eriksen R, Astrup R and Øyen B-H (2012) Effekter av ulike miljøhensyn på tilgjengelig skogareal og volum i norske skoger. Rapport fra Skog og Landskap 2/2012. Ås: Skog og Landskap.

9. Gjerde I and Baumann C (2002) Miljøregistrering i skog - biologisk mangfold. Ås: Norwegian Institute for Forest Research.

10. Game ET and Grantham HS (2008) Marxan User Manual: For Marxan version 1.8.10. St. Lucia, Queensland, Australia, Vancouver, BC, Canada: University of Queensland, Pacific Marine Analysis and Research Association.

11. Blumentrath S, Bergseng E, Astrup R and Barton DN (2013) Using National Forest Inventories and publicly available map data for geographical mapping of opportunity costs of forestry environmental considerations. . In: H. Lindhjem, D. N. Barton, G. M. Rusch, A. Sverdrup‐Thygeson and S. Blumentrath, editors. Assessment of impact of proposed policy instruments for biodiversity conservation at local/municipal level – fine grain analysis for Norway POLICYMIX Report Del 712. Oslo: NINA.

12. Bergseng E, Ask JA, Framstad E, Gobakken T, Solberg B, et al. (2012) Biodiversity protection and economics in long term boreal forest management — A detailed case for the valuation of protection measures. For Policy Econ: 12-21.
